# ﻿Four new species of *Perilimnastes* (Sonerileae, Melastomataceae) from Vietnam

**DOI:** 10.3897/phytokeys.235.112133

**Published:** 2023-11-03

**Authors:** Jin-Hong Dai, Truong Van Do, Ying Liu

**Affiliations:** 1 School of Life Sciences, Sun Yat-sen University, Guangzhou 510275, China; 2 State Key Laboratory of Biocontrol and Guangdong Key Laboratory of Plant Resources, Sun Yat-sen University, No. 135, Xin-Gang-Xi Road, Guangzhou 510275, China; 3 Vietnam National Museum of Nature, Vietnam Academy of Science and Technology, 18th Hoang Quoc Viet Road, Cau Giay, Hanoi, Vietnam; 4 Graduate University of Science and Technology, Vietnam Academy of Science and Technology, 18th Hoang Quoc Viet Road, Cau Giay, Hanoi, Vietnam; 5 School of Ecology, Sun Yat-sen University, Shenzhen 518107, China

**Keywords:** Melastomataceae, *Perilimnastes*, *Phyllagathis*, taxonomy

## Abstract

*Perilimnastes* is a genus currently treated in the polyphyletic *Phyllagathis*. Recent phylogenomic analyses have identified a morphologically cohesive lineage referred to as the *Phyllagathis* (raphides) clade, which should be excluded from *Phyllagathis* and treated as a distinct genus under the name *Perilimnastes*. Morphological and phylogenomic data have confirmed that four new species collected from Vietnam are part of the *Phyllagathis* (raphides) clade. They are described herein as *Perilimnastesmultisepala*, *P.setipetiola*, *P.uniflora*, and *P.banaensis*. *Perilimnastesmultisepala* is phylogenetically closest to *Phyllagathissetotheca*, and morphologically to *P.fruticosa* and *P.stenophylla*, but is distinct in the 4- to 8-lobed calyx, 28 × 9 mm, apically long acuminate petals, and 1–2 mm pedicel at fruiting stage. *Perilimnastessetipetiola*, *P.uniflora*, and *P.banaensis* are phylogenetically most closely related. *Perilimnastesuniflora* is characterized by its prostrate habit, small size, glabrous, obovate to obovate-lanceolate leaf blade, and solitary flower. *Perilimnastessetipetiola* and *P.banaensis* resemble each other in habit, leaf size and shape, and sessile or near sessile inflorescences but can be easily distinguished by the indumentum of the stems and leaves.

## ﻿Introduction

*Perilimnastes* Ridl. was established based on *P.fruticosa* (Ridl.) Ridl. (Ridley 1918, 1922), a species originally published in *Anerincleistus* Korth. as *A.fruticosus* Ridl. (Ridley 1908). *Perilimnastesfruticosa* is characterized by its shrubby habit, subcoriaceous 3-veined leaves, few-flowered cymes, isomorphic stamens, and crowned capsules. It grows on rocks along streams in forests. Ridley (1918) noted that the fruit of *P.fruticosa* (=*A.fruticosus*) did not fit into any existing genera and described it as a distinct genus named *Perilimnastes*. Nayar (1974) accepted Ridley’s concept of *Perilimnastes* and described a second species, *P.rupicola* M.P.Nayar, which resembles *P.fruticosa* in habit and morphology of leaves, calyx lobes, stamens, and capsules. However, subsequent authors didn’t recognize *Perilimnastes*. Maxwell (1982, 1989) synonymized *Perilimnastes* and accommodated its two members in Anerincleistussect.Coriaceae Ridl. (as *A.fruticosus*) and sect. Anerincleistus [as *A.rupicola* (Nayar) J.F.Maxwell], respectively. On the other hand, Cellinese (2002, 2003) placed both species within the broadly defined *Phyllagathis* Blume [as *P.fruticosa* (Ridl.) C.Hansen ex Cellin. and *P.rupicola* (M.P.Nayar) C.Hansen ex Cellin., respectively].

The classification of Asian Sonerileae at generic level has been a topic of ongoing controversy, particularly regarding the delimitation of *Phyllagathis* and various genera morphologically related to it (Diels 1932; Li 1944; Chen 1984a, b; Hansen 1992; Cellinese and Renner 1997; Cellinese 2002, 2003; Chen and Renner 2007). A series of molecular phylogenetic analyses consistently demonstrated the polyphyletic nature of *Phyllagathis* (Zeng et al. 2016; Zhou et al. 2018; Zhou et al. 2019a, b, c; Liu et al. 2022; Zhou et al. 2022). Zhou et al. (2022) presented the first well-resolved phylogeny of Asian Sonerileae and identified 34 major clades based on genome-scale data. Species currently treated in *Phyllagathis* were found in 17 lineages scattered across Asian Sonerileae. The type of *Phyllagathis* showed no close relationships with other members and the genus may have to be redefined as monotypic. Samples of *Anerincleistus* formed a strongly supported clade with certain Bornean species of *Phyllagathis*. The generic type of *Perilimnastes*, namely *P.fruticosa* (also known as *Phyllagathisfruticosa* and *Anerincleistusfruticosus*), was not included in the analyses. Nonetheless, species closely resembling *P.fruticosa*, such as *Phyllagathisstenophylla* (Merr. & Chun) H.L.Li and *Phyllagathissuberalata* C.Hansen, were recovered as part of the *Phyllagathis* (raphides) clade, which consists of members characterized by a fruticose/suffruticose growth habit, cuneate to rounded leaf base, umbellate or cymose inflorescences sometimes sessile or reduced to a single flower, isomorphic stamens, crowned capsules, horned placental column, thready placentas, as well as the presence of raphide crystals in some species. Based on these diagnostic characteristics and notable similarity observed between sampled and unsampled species, Zhou et al. (2022) concluded that the *Phyllagathis* (raphides) clade should contain approximately 20 species distributed in southernmost China, Vietnam, the Malay Peninsula, and Borneo. Given its distant relationship with the generic type of *Phyllagathis*, this clade justifies recognition as a distinct genus. As compelling morphological evidence indicates that the type of *Perilimnastes* (*P.fruticosa*) is a member of the *Phyllagathis* (raphides) clade, *Perilimnastes* should be resurrected as its generic name (Zhou et al. 2022).

**Figure 1.**
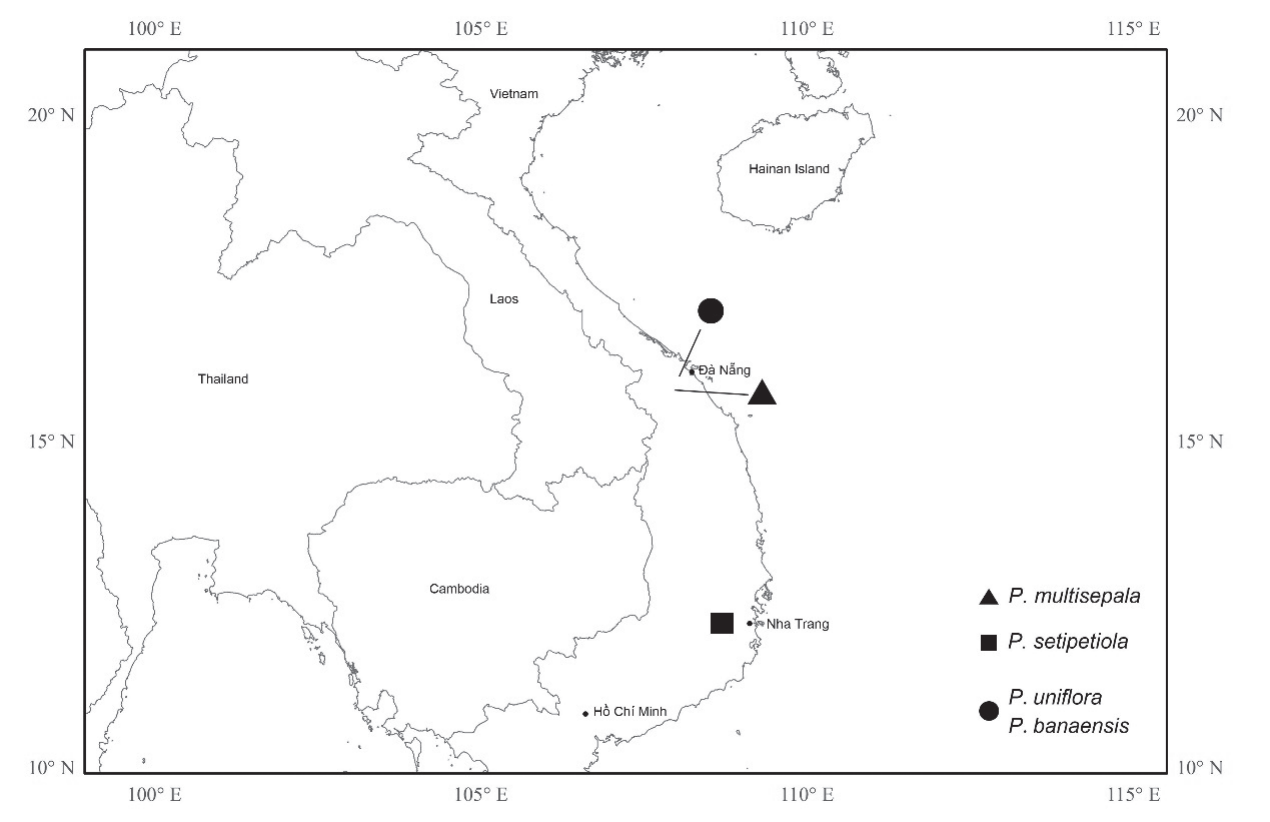
Distribution of *Perilimnastesmultisepala* (triangle), *P.setipetiola* (square), and *P.uniflora* and *P.banaensis* (solid circle).

**Figure 2.**
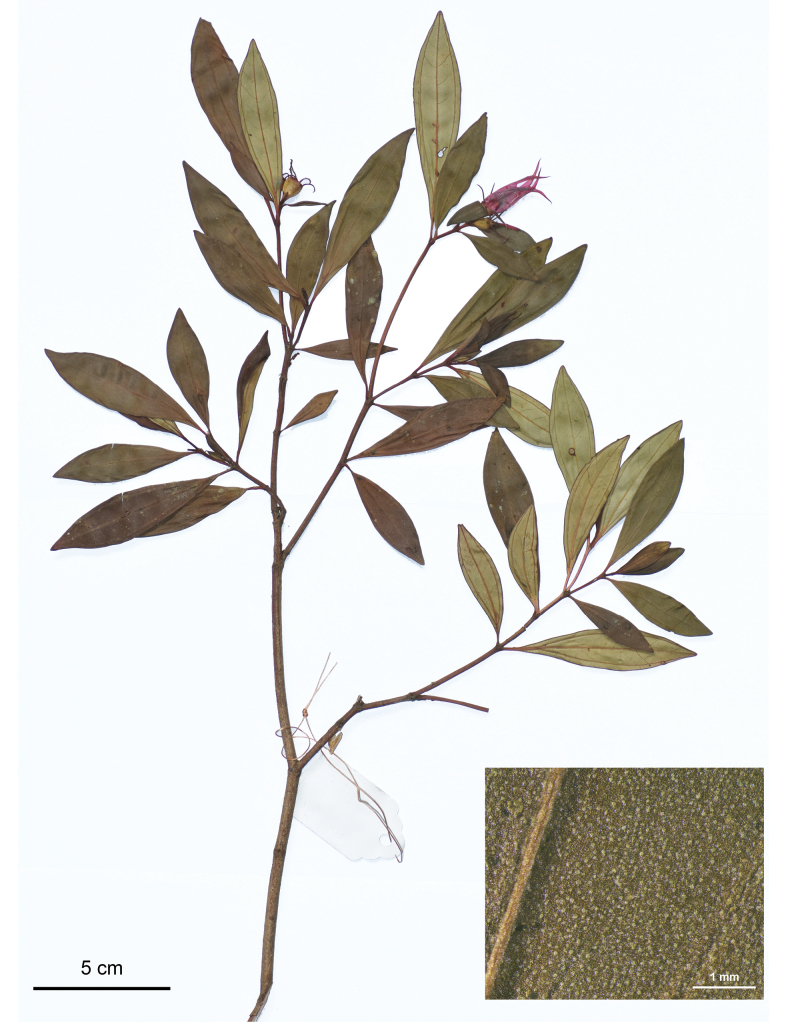
Holotype of *Perilimnastesmultisepala*, Jin-hong Dai and Ying Liu 821 (PE). The inset shows druses (as white spots) on adaxial leaf surface under stereoscope. Scale bars: 5 cm, 1 mm (inset).

**Figure 3.**
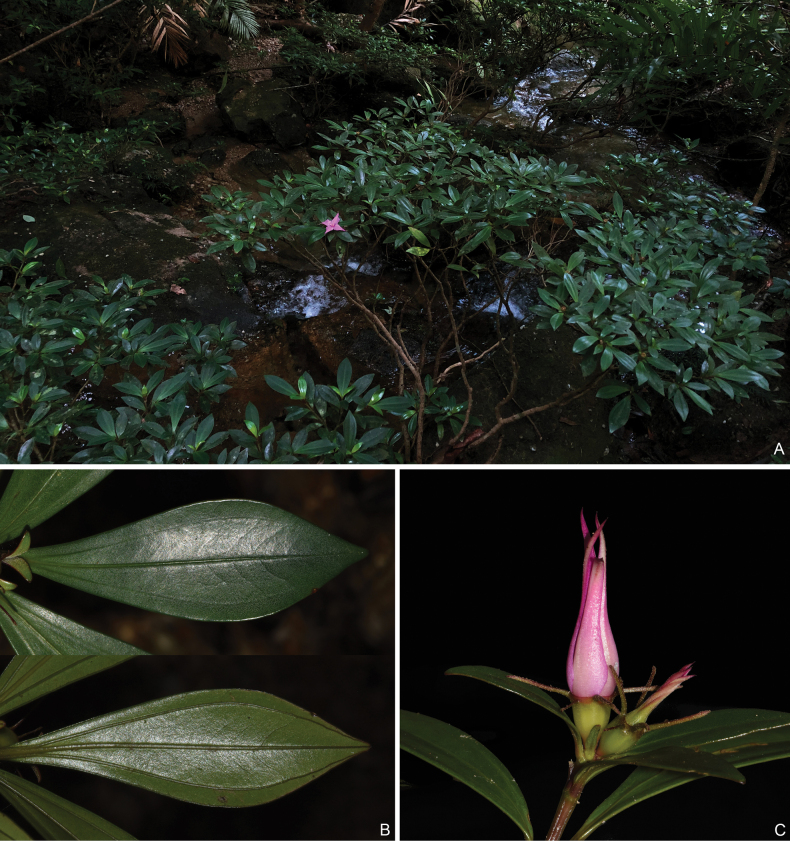
*Perilimnastesmultisepala***A** habit **B** adaxial (top) and abaxial (bottom) leaf surfaces **C** close-up of a branchlet showing a 2-flowered cyme. All from Jin-hong Dai and Ying Liu 821 (A, PE, SYS).

**Figure 4.**
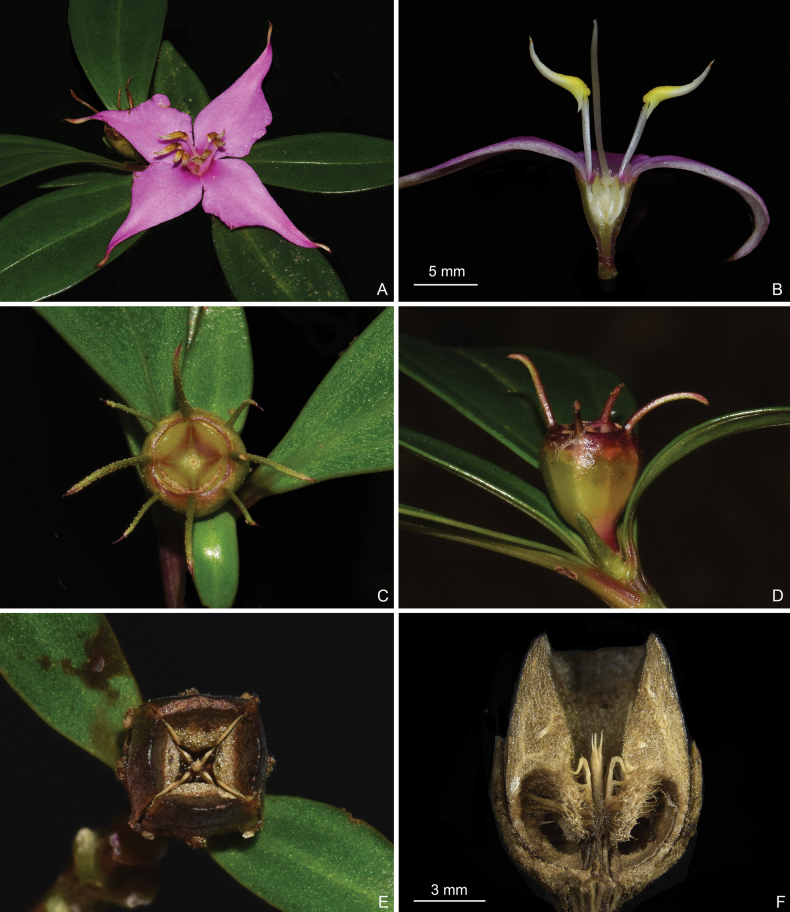
*Perilimnastesmultisepala***A** top view of a flower **B** longitudinal section of a flower showing the isomorphic stamens **C** top view of a young capsule **D** lateral view of a young capsule **E** top view of an old capsule **F** longitudinal section of an old capsule showing enlarged ovary crown and morphology of the placental column and placentas. Scale bars: 5 mm (**B**); 3 mm (**F**). All from Jin-hong Dai and Ying Liu 821 (A, PE, SYS).

**Figure 5.**
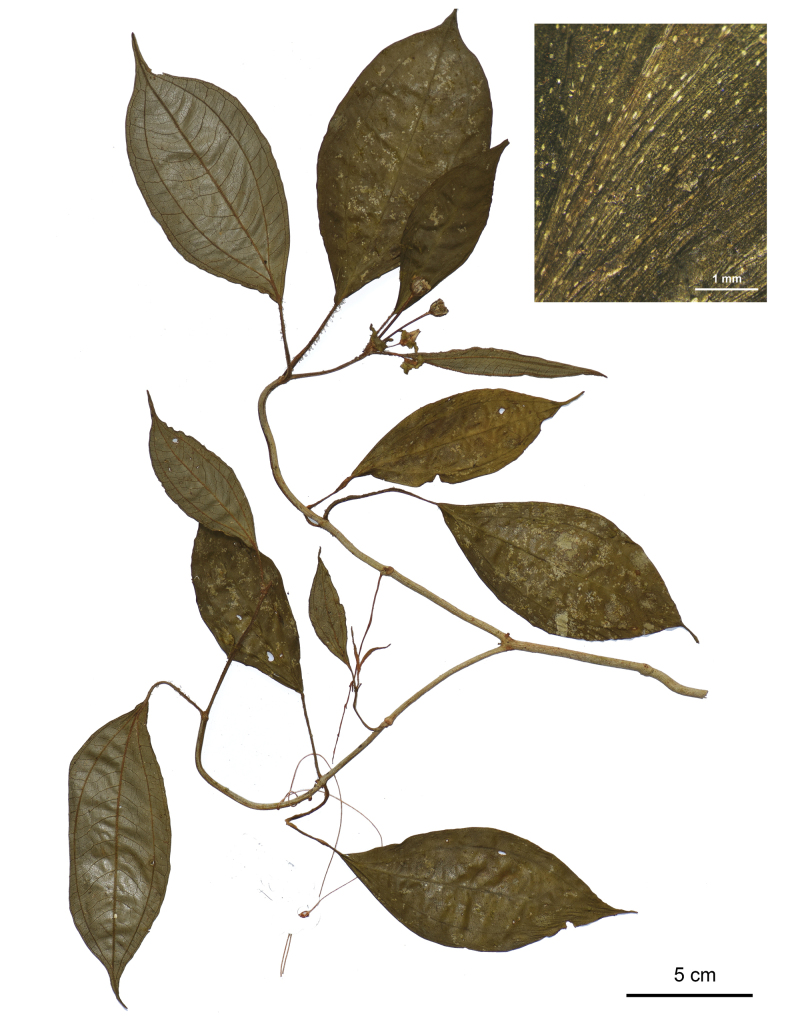
Holotype of *Perilimnastessetipetiola*, Jin-hong Dai and Ying Liu 836 (PE). The inset shows raphides (as white oblong spots) on adaxial leaf surface under stereoscope. Scale bars: 5 cm, 1 mm (inset).

**Figure 6.**
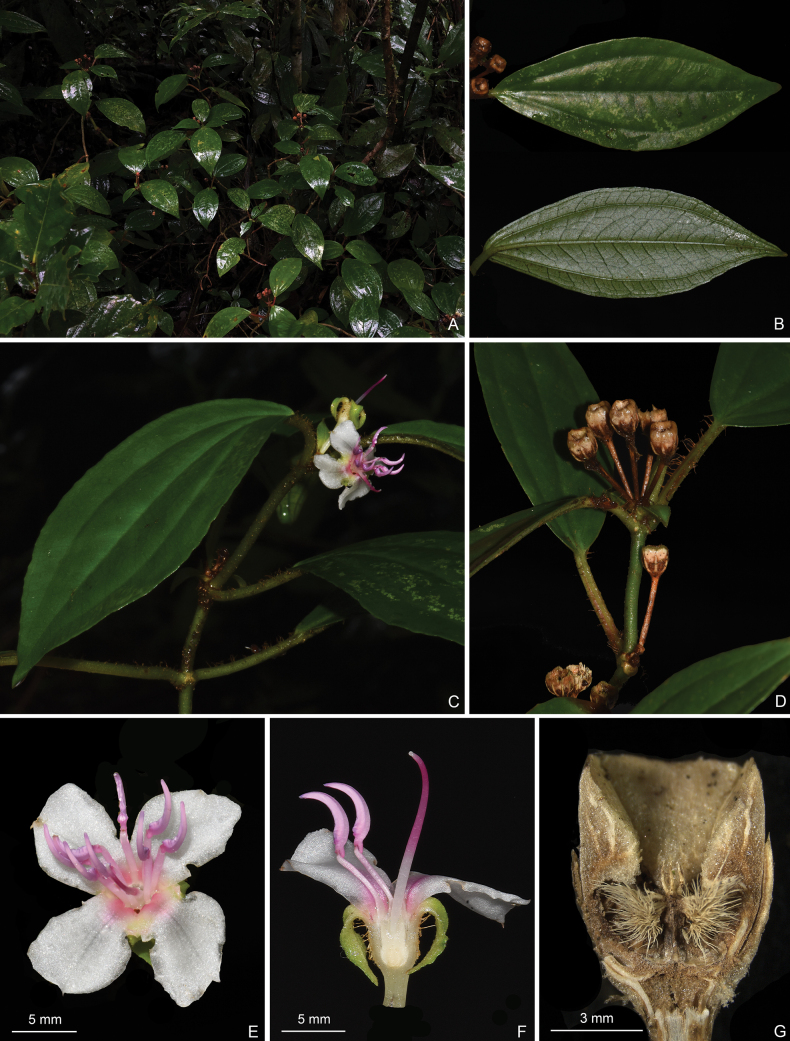
*Perilimnastessetipetiola***A** habit **B** adaxial (top) and abaxial (bottom) leaf surfaces **C** a flowering branch **D** a branch showing hispid petioles and terminal and axillary infructescences **E** top view of a flower **F** longitudinal section of a flower showing the isomorphic stamens **G** longitudinal section of an old capsule showing enlarged ovary crown and morphology of the placental column and placentas. Scale bars: 5 mm (**E**, **F**); 3 mm (**G**). All from Jin-hong Dai and Ying Liu 836 (A, PE, SYS).

**Figure 7.**
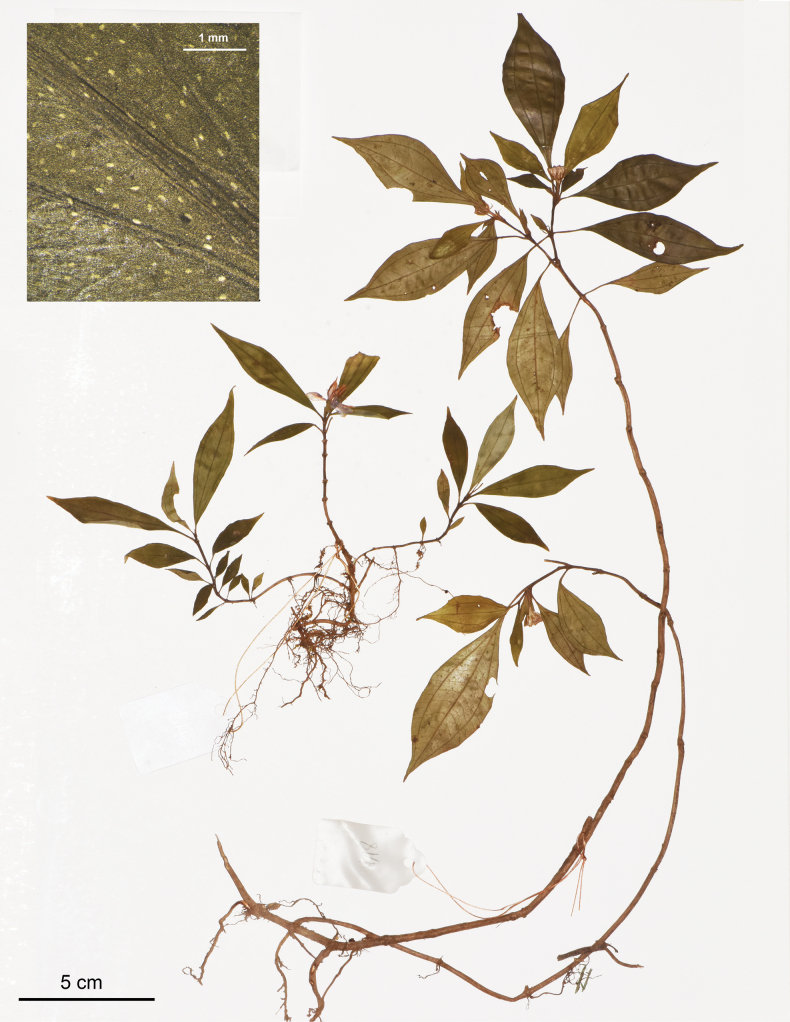
Holotype of *Perilimnastesuniflora*, Jin-hong Dai and Ying Liu 814 (PE). The inset shows raphides (as white oblong spots) on adaxial leaf surface under stereoscope. Scale bars: 5 cm, 1 mm (inset).

**Figure 8.**
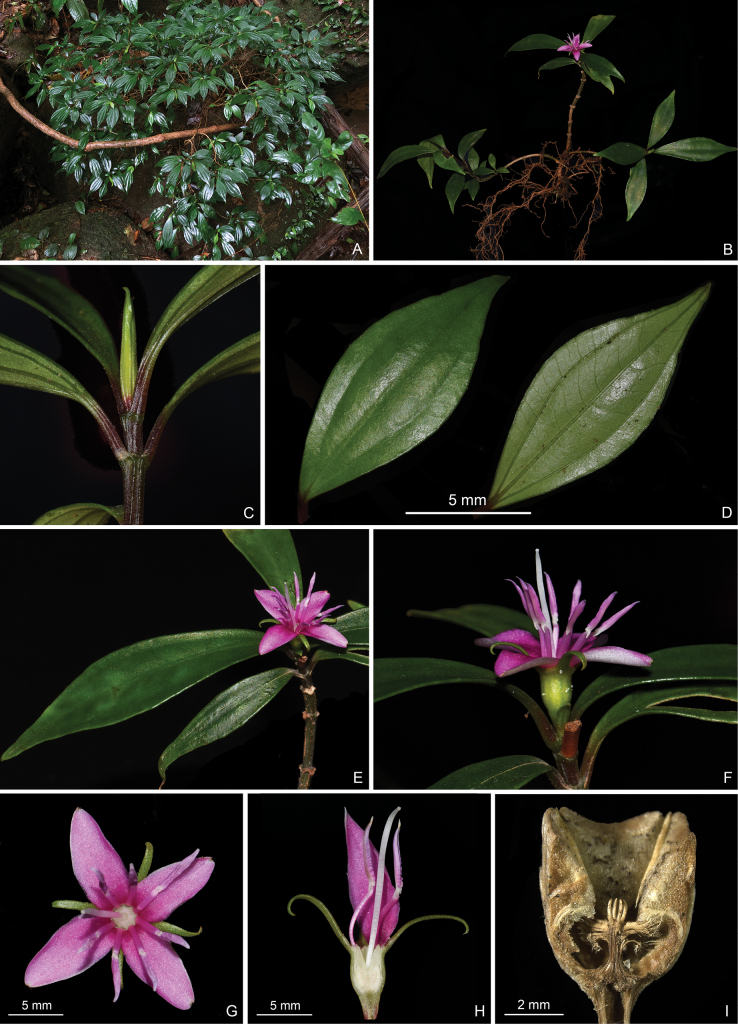
*Perilimnastesuniflora***A** habit **B** a flowering individual **C** close-up of a branchlet **D** adaxial (left) and abaxial (right) leaf surfaces **E** a flowering branch **F** close-up of an inflorescence showing a solitary flower **G** top view of a flower **H** longitudinal section of a flower showing the isomorphic stamens **I** longitudinal section of an old capsule showing enlarged ovary crown and morphology of the placental column and placentas. Scale bars: 5 mm (**D**, **G**, **H**); 2 mm (**I**). All from Jin-hong Dai and Ying Liu 814 (A, PE, SYS).

**Figure 9.**
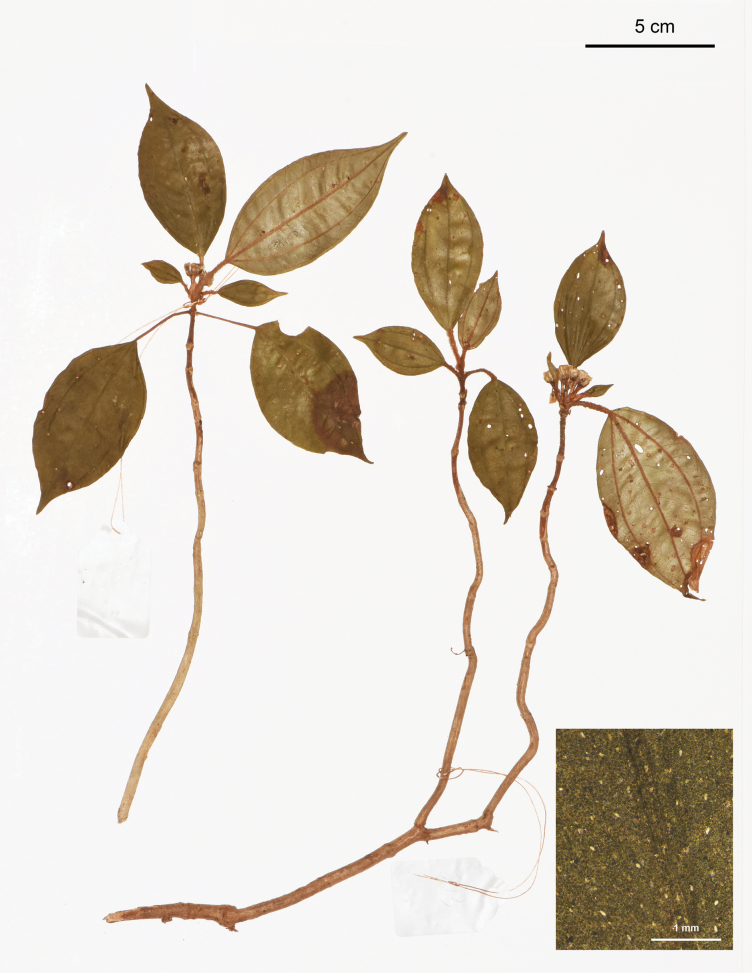
Holotype of *Perilimnastesbanaensis*, Jin-hong Dai and Ying Liu 813 (PE). The inset shows raphides (as white oblong spots) on adaxial leaf surface under stereoscope. Scale bars: 5 cm, 1 mm (inset).

**Figure 10.**
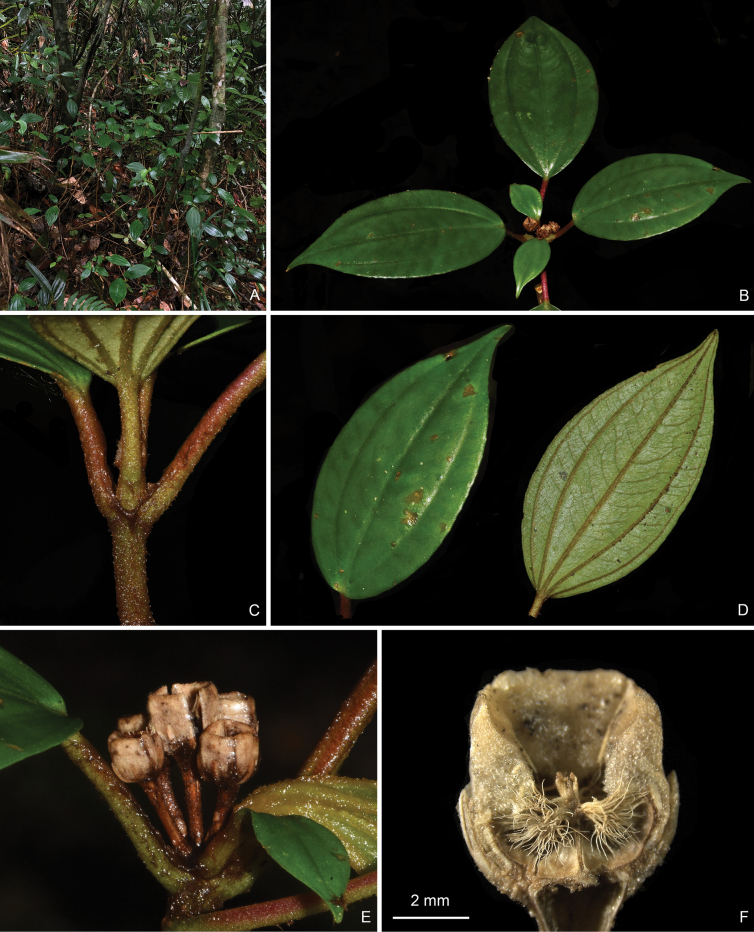
*Perilimnastesbanaensis***A** habit **B** a branch with old capsules **C** close-up of a branchlet **D** adaxial (left) and abaxial (right) leaf surfaces **E** a sessile infructescence **F** longitudinal section of an old capsule showing enlarged ovary crown and morphology of the placental column and placentas. Scale bars: 2 mm (**F**). All from Jin-hong Dai and Ying Liu 813 (A, PE, SYS).

During a field expedition in Vietnam, four species that were previously unrecorded in the Flora of Vietnam were collected from Đại Lộc, Quảng Nam Province (1 sp.), Đà Lạt, Lâm Đồng Province (1 sp.), and Hòa Ninh, Đà Nẵng (2 spp.) (Fig. 1). These plants share strong morphological resemblance to *Perilimnastes* [= the *Phyllagathis* (raphides) clade] and their placement within this clade was later confirmed through phylogenomic analyses conducted by Zhou et al. (2022). Morphological comparison between the four plants and their possible relatives revealed that they represent species new to science, which we described below as *Perilimnastesmultisepala* J.H.Dai, T.V.Do & Ying Liu (Figs 2–4), *P.setipetiola* J.H.Dai, T.V.Do & Ying Liu (Figs 5, 6), *P.uniflora* J.H.Dai, T.V.Do & Ying Liu (Figs 7, 8), and *P.banaensis* J.H.Dai, T.V.Do & Ying Liu (Figs 9, 10).

## ﻿Morphological comparison

Morphological and distribution data were obtained through field, herbarium and literature surveys as well as observation of living plants in the facilities of Sun Yat-sen University. Specimens of the species concerned (GXMI, IBSC, IBK, KUN, PE, SYS) or their high-resolution photos (A, BM, C, E, G, K, NY, P, US) were examined. Species delimitation followed Chen (1984b), Hansen (1992), and Cellinese (2002, 2003).

According to previous phylogenomic analyses, *P.multisepala* is closest to *Phyllagathissetotheca* H.L.Li from China (Zhou et al. 2022). However, the two species are quite distinct in leaf size (2.4–8 × 0.7–2.4 cm vs. 10–20 × 3–8 cm), number of flowers per inflorescence (1 or 2, rarely 3 vs. 3 to more than 20), number and shape of calyx lobes (4–8, linear vs. 4, long triangular) and length of pedicels (1–2 mm vs. 8–18 mm). In terms of morphology, *P.multisepala* closely resembles *P.fruticosa* and *P.stenophylla*. All three species are shrubs with druses, somewhat oblong-lanceolate, coriaceous, 3-veined leaf blade, few-flowered inflorescences, and narrow calyx lobes. Nevertheless, *P.multisepala* can be easily distinguished from the latter two species in the petals 28 × 9 mm, apically long acuminate (vs. 8.5–16 × 3.5–5 mm, acuminate, and 12 × 6 mm, short acuminate), calyx lobes 4–8 (vs. 4) and pedicels 1–2 mm at fruiting stage (vs. 10–15 mm). A comparison of the four species can be found in Suppl. material 1: table S1.

*Perilimnastessetipetiola* is resolved as sister to *P.uniflora*, and *P.setipetiola*-*P.uniflora* to *P.banaensis* (Zhou et al. 2022). Despite their close relationship, these species are morphologically quite different from one another. *Perilimnastesuniflora* is characterized by its small size (to 30 cm tall), the whole plant glabrous except for sparse minute brown glands when young, stems prostrate at middle and lower parts, leaf blade obovate to obovate-lanceolate, and solitary flower. *Perilimnastessetipetiola* and *P.banaensis* resemble each other in the shrubby habit, leaf size, somewhat elliptic leaf blade, and sessile or subsessile inflorescences with multiple flowers, however, the two species differ markedly in the indumentum of the stems and petioles (stems and petioles pubescent with stellate hairs when young, petioles hispid with stout, 2–4 mm long bristles vs. densely villous with appressed brown hyaline uniseriate hairs). According to Zhou et al. (2022), *P.setipetiola*, *P.uniflora*, and *P.banaensis* formed the sister clade of a Bornean lineage containing *Phyllagathiselliptica* Stapf and *P.dispar* (Cogn.) C.Hansen. The former three species are linked to the Bornean lineage by the presence of raphide crystals, somewhat elliptic leaf blade, and terminal and axillary umbels with very short or no peduncles in some of the species, however, they can be distinguished from the latter based on a combination of height, habit, indumentum, and anther morphology. In addition to the Bornean lineage, *P.setipetiola* shares similarities in habit, leaf size and shape with *P.setotheca* and *P.ovalifolia* H.L.Li. However, it differs from the latter species in terms of the indumentum of leaf petiole and the length of peduncles. *Perilimnastesuniflora* also resembles *Phyllagathisguillauminii* H.L.Li and *P.rupicola*, two species not sampled in previous phylogenetic studies, in crystal type and leaf shape, but differs in indumentum and the length of the pedicel at fruiting stage. A comparison of the species discussed above is provided in Suppl. material 1: table S2.

Phylogenetic data and morphological comparison justify the recognition of *P.multisepala*, *P.setipetiola*, *P.uniflora*, and *P.banaensis* as distinct species in *Perilimnastes*. The formal taxonomic treatment of other species in the *Phyllagathis* (raphides) clade will be dealt with in another study.

## ﻿Geographical distribution

The four new species are geographically quite isolated from related species previously discussed (Suppl. material 1: tables S1, S2). *Perilimnastesmultisepala* is located in central Vietnam (Fig. 1), whereas its related species, *P.setotheca* and *P.stenophylla* are documented in southernmost China and northern Vietnam, and *P.fruticosa* in the Malay Peninsula. *Perilimnastessetipetiola* is distributed in Đà Lạt, southern Vietnam and *P.banaensis* and *P.uniflora* are found in central Vietnam (Fig. 1). The three species are morphologically/phylogenetically related to *P.elliptica*, *P.dispar*, *P.rupicola*, *P.setotheca*, *P.ovalifolia*, and *P.guillauminii*. *Phyllagathiselliptica*, *P.dispar*, and *P.rupicola* are endemic species of Borneo, *P.setotheca* and *P.ovalifolia* are found in southernmost regions of China and northern Vietnam, while *P.guillauminii* has been documented in Bien Hoa, southern Vietnam. Members of the *Phyllagathis* (raphides) clade typically inhibit moist and shady environments in forests, such as damp slopes or rocky areas along or near streams and waterfalls. However, it is uncommon for multiple species of this clade to coexist in the same location. In this particular case, only *P.banaensis* and *P.uniflora* were observed together at an elevation of 1,360 m near the summit of Ba Na Hills in central Vietnam. Nevertheless, the two species prefer somewhat different microhabitats. Individuals of *P.banaensis* occupy damp slopes alongside other shrubs and lianas, whereas those of *P.uniflora* typically inhabit moist exposed rocks with fewer shrubs and lianas around.

## ﻿Taxonomic treatment

### ﻿ Perilimnastes multisepala

Taxon classificationPlantaeMyrtalesMelastomataceae

J.H.Dai, T.V.Do & Ying Liu sp. nov.

48251918-F33D-5760-BF8D-A2895F2356A8

urn:lsid:ipni.org:names:77329901-1

Figs 2 , 3 , 4

#### Type.

Vietnam. Quảng Nam Province: Đại Lộc, about 400 m south of Khu Du Lich Sinh Thai Khe Lim, along newly opened road, 574 m elevation, on rocks along a stream, 23 Nov 2019, Jin-hong Dai and Ying Liu 821 (holotype: PE; isotypes: A, SYS, VNMN).

#### Diagnosis.

Resembles *P.fruticosa* and *P.stenophylla* in the habitat preference, habit, leaf and inflorescence morphology but differs from these species in the petals 28 × 9 mm, apex long acuminate (vs. 8.5–16 × 3.5–5 mm, acuminate, and 12 × 6 mm, short acuminate), calyx lobes 4–8 (vs. 4) and pedicels only 1–2 mm at fruiting stage (vs. 10–15 mm).

#### Description.

Shrubs, much-branched, up to 0.8 m tall, with druses in many parts. Stems obtusely 4-sided, slightly compressed when young; branchlets glabrous, sulcate, nodes only pubescent with uniseriate hairs when young. Leaves opposite, equal to distinctly unequal in a pair, pubescent with brownish-yellow stellate hairs only when young, glabrous when mature; petiole 0–10 mm; leaf blade obovate-lanceolate, oblong-lanceolate to oblanceolate, 2.4–8 × 0.7–2.4 cm, subcoriaceous, 3-veined with the lateral two veins often diverged from the midvein above the base, dark green adaxially, pale green abaxially, base cuneate, margin entire, apex obtuse, acute, rarely shortly acuminate. Inflorescences terminal, cymose contracted to umbellate, solitary or 2-flowered, rarely 3-flowered; peduncle ca. 1 mm long, sometimes sessile, subtended by a pair of bracts to 5 mm long. Flowers 4-merous; pedicel 1–2 mm long, glabrous; hypanthium funnel-shaped, 7–8 mm long, sparsely pubescent with stellate hairs; calyx lobes linear, laterally compressed, alternipetalous 4, 8–10 mm long, antepetalous 0–4, 3–8 mm long, sparsely pubescent with stellate hairs; petals pinkish purple, 28 × 9 mm, ovate, slightly oblique, apex long acuminate, abaxially very sparsely pubescent with stellate hairs; stamens 8, isomorphic, filaments 7–9 mm long, glabrous, anthers lanceolate, yellow, 9 mm long, connective decurrent, tuberculate ventrally, forming a spur dorsally; ovary half as long as hypanthium (crown excluded), ovary crown wedge-like, 4-lobed; style 22 mm long. Capsule cup-shaped, 7–8 × 7 mm, 4-sided; hypanthium 8-ribbed; crown enlarged enclosing an obpyramidal space; placental column unbeaked, 4-horned; placenta thready.

#### Phenology.

Flowers, young fruits and old fruits in November.

#### Etymology.

The specific epithet is based on the 4–8 calyx lobes of this species.

#### Distribution.

*Perilimnastesmultisepala* is currently known from Đại Lộc, Quảng Nam Province, Vietnam (Fig. 1). It grows on rocks along streams in the forest, at 574 m elevation.

### ﻿ Perilimnastes setipetiola

Taxon classificationPlantaeMyrtalesMelastomataceae

J.H.Dai, T.V.Do & Ying Liu sp. nov.

F991CE9C-5CD6-56B8-AA2F-4D345875AAF0

urn:lsid:ipni.org:names:77329904-1

Figs 5 , 6

#### Type.

Vietnam. Lâm Đồng Province: Đà Lạt, Bidoup Nui Ba National Park, 1,500–1,700 m elevation, at damp places under forest, 29 Nov 2019, Jin-hong Dai and Ying Liu 836 (holotype: PE; isotypes: A, SYS, VNMN).

#### Diagnosis.

Resembles *P.banaensis*, *P.elliptica* and *P.dispar* in having hyaline hairs, raphide crystals, somewhat elliptic leaf blade and umbels with very short or no peduncles but differs markedly from *P.banaensis* in the indumentum of the stems and petioles (both pubescent with stellate hairs when young, petioles hispid with long bristles vs. densely villous with appressed hyaline uniseriate hairs), and from the latter two species in height (40–120 cm vs. up to 45 cm), habit (shrubby vs. herbal), anther color (pink vs. yellow) and the morphology of connectives (prolonged below anthers vs. not prolonged). Also resembles *P.setotheca* and *P.ovalifolia* in habit, leaf size and shape but differs in petiole hispid with stout, 2–4 mm long bristles (vs. glabrous in *P.setotheca* and densely hirsute with soft hairs in *P.ovalifolia*) and umbels with 0–2 mm peduncles (vs. peduncles 8–18 mm long in *P.setotheca* and 10–30 mm long in *P.ovalifolia*).

#### Description.

Shrubs, 40–120 cm tall, branched, with raphides in all parts. Stems obtusely 4-sided when young, pubescent with brownish-yellow stellate hairs and rarely uniseriate hyaline hairs (both composed of elongated cells) when young, glabrescent when mature. Leaves opposite, equal or subequal in a pair; petiole 1.2–6 cm long, pubescent with brownish-yellow stellate hairs when young, hispid with stout, 2–4 mm long bristles; leaf blade broadly elliptic to elliptic, 5.6–15 × 1.9–6.4 cm, papery to stiffly papery, pubescent with brownish-yellow stellate hairs when young, glabrous on the upper surface and sparsely pubescent along veins on lower surface when mature, often 5-veined with the marginal two slightly inconspicuous and the inner two diverged from the midvein above the base, base cuneate, margin entire, apex acuminate, short acuminate, rarely acute. Inflorescences terminal and axillary, umbellate, 2–11-flowered, subtended by two sessile bracts; sessile or with peduncle up to 2 mm long. Flowers 4-merous; pedicel 8–13 mm long (16–25 mm in fruit), glabrous; hypanthium funnel-shaped, 5–7 mm long, pubescent with multiseriate hairs and sparsely so with stellate hairs; calyx lobes triangular-ovate, 6 mm long, glabrescent; petals pinkish-white, broadly ovate, oblique, ca. 10 mm long, apex acute; stamens isomorphic, filaments ca. 6 mm, anthers pink, lanceolate, ca. 6 mm, connective decurrent, prolonged below anther, forming a spur dorsally; ovary half as long as hypanthium (crown excluded), ovary crown wedge-like, 4-lobed; style 15 mm long. Capsule cup-shaped, ca. 7 × 6 mm, 4-sided; hypanthium 8-ribbed; crown enlarged enclosing an obpyramidal space; placental column unbeaked, 4-horned; placenta thready.

#### Phenology.

Flowers and old fruits in November.

#### Etymology.

The specific epithet is based on the stout long bristles on the petiole of this species.

#### Distribution.

*Perilimnastessetipetiola* is currently known from Đà Lạt, Lâm Đồng Province, Vietnam (Fig. 1). It occurs at damp places in forests, at 1,500–1,700 m elevation.

#### Additional specimen examined.

Vietnam. Lâm Đồng Province: Lạc Dương district, 40 km to northeast from Đà Lạt city. Closed primary wet broadleaved cloud forest on southwest macroslope of Hon Giao mountain ridge at 1,600–1,700 m elevation, 21 Apr 1997, L.Averyanov, N.Q.Binh, N.T.Hiep, VH 4133 [P (P05200269)].

### ﻿ Perilimnastes uniflora

Taxon classificationPlantaeMyrtalesMelastomataceae

J.H.Dai, T.V.Do & Ying Liu sp. nov.

C4B10E66-EB2C-5BC9-814A-EB76D73B664D

urn:lsid:ipni.org:names:77329905-1

Figs 7 , 8

#### Type.

Vietnam. Đà Nẵng: Hòa Ninh, Ba Na Hills, 1,360 m elevation, in forests on damp rocks along steam, 22 Nov 2019, Jin-hong Dai and Ying Liu 814 (holotype: PE; isotypes: A, SYS, VNMN).

#### Diagnosis.

Resembles *P.guillauminii* and *P.rupicola* in having raphide crystals, 3-veined leaves with cuneate base and somewhat acuminate apex, and narrow calyx lobes, but differs from *P.rupicola* in its pink anthers (vs. yellow) and from both in the stems glabrous except for minute brown glands when young (vs. covered with long bristles in *P.guillauminii* and hyaline uniseriate hairs in *P.rupicola*) and pedicel 4 mm long at fruiting stage (vs. 16 mm long in *P.guillauminii* and 22 mm long in *P.rupicola*).

#### Description.

Shrublets or somewhat woody herbs, to 30 cm tall, with raphides in all parts. Stems prostrate at middle and lower parts, branched, with adventitious roots at lower nodes; branchlets quadrangular and with sparse minute brown glands when young, glabrescent; older branches obtusely 4-sided; leafy distally and leafless proximally. Leaves opposite, equal to unequal in a pair, with minute brown glands only when young, glabrescent when mature; petiole 0.5–2 cm long; leaf blade obovate to obovate-lanceolate, sometimes elliptic, 4.2–9.5 × 1.3–3.4 cm, papery, 3-veined with the lateral two veins diverged from the midvein at or above the base, green to dark green adaxially, pale green abaxially, base cuneate to narrowly cuneate, margin entire or inconspicuous minutely repand, apex acuminate to long acuminate, sometimes caudate. Inflorescences terminal, flower solitary, subtended by a pair of bracts ca. 4 mm long. Flowers 4-merous; pedicel ca. 2 mm long, 4 mm at fruiting stage, glabrous; hypanthium funnel-shaped, 5–6 mm long, glabrous except for minute glands; calyx lobes 4, linear, 6–9 mm long, with minute glands; petals pinkish-purple, 11–13 × 4–6 mm, ovate, minutely oblique, apex acute to short acuminate, glabrous on both sides; stamens 8, isomorphic, filaments pink, ca. 6 mm long, glabrous, anthers lanceolate, pink, 5–7 mm long, connective decurrent, forming two ventral lobes and a dorsal spur; ovary half as long as hypanthium (crown excluded), ovary crown wedge-like, 4-lobed; style 13–15 mm long. Capsule cup-shaped, ca. 5 × 5 mm, 4-sided; hypanthium 8-ribbed; crown enlarged enclosing an obpyramidal space; placental column unbeaked, 4-horned; placenta thready.

#### Phenology.

Flowers in June and produces old fruits in November.

#### Etymology.

The specific epithet is based on the solitary flowers of this species.

#### Distribution.

*Perilimnastesuniflora* is currently only known from Ba Na Hills, Hòa Ninh, Đà Nẵng, Vietnam (Fig. 1). It occurs on damp rocks along streams in forests, at 1,360 m elevation.

### ﻿ Perilimnastes banaensis

Taxon classificationPlantaeMyrtalesMelastomataceae

J.H.Dai, T.V.Do & Ying Liu sp. nov.

260EAE91-88AE-55FE-BFEA-E1E726111BA9

urn:lsid:ipni.org:names:77329906-1

Figs 9 , 10

#### Type.

Vietnam. Đà Nẵng: Hòa Ninh, Ba Na Hills, 1,360 m elevation, in forests on damp slopes near steam, 22 Nov 2019, Jin-hong Dai and Ying Liu 813 (holotype: PE; isotypes: A, SYS, VNMN).

#### Diagnosis.

Resembles *P.ovalifolia* and *P.setipetiola* in having raphide crystals, hyaline hairs, somewhat elliptic leaf blade, and umbels with very short or no peduncle, but differs in the stems and petioles densely villous with brown hyaline uniseriate hairs (vs. stems densely retrorse hirsute, glabrescent, and petioles densely hirsute to setose in the former species, and stems pubescent with brownish-yellow stellate hairs and rarely also hyaline hairs, petioles with brownish-yellow stellate hairs when young and hispid with long bristles in the latter).

#### Description.

Shrubs to 60 cm tall, with raphides in all parts. Stems branched, prostrate at lower parts; branchlets obtusely 4-sided and densely villous with appressed, brown hyaline uniseriate hairs composed of much elongated cells and tipped with a brown glandular cell; older branches near terete and glabrescent; leafy distally and leafless proximally. Leaves opposite, equal to unequal in a pair; petiole 1.5–4.6 cm long, densely villous with appressed, brown hyaline hairs; leaf blade elliptic, 5.5–13 × 2.5–6.5 cm, thick papery, with minute brown glands when young on both surfaces, abaxially sparsely pubescent with appressed brown hyaline hairs, densely so along the veins, 3 or 5-veined, dark green adaxially, pale green abaxially, base acute to rounded, margin entire or sometimes inconspicuously minutely repand, apex short acuminate to acute. Inflorescences and flowers unknown. Infructescences terminal, umbellate, sessile, capsules 2–7, pedicel 5–13 mm long. Old capsules cup-shaped, ca. 5 × 5 mm, 4-sided; hypanthium 8-ribbed; crown enlarged enclosing an obpyramidal space; placental column unbeaked, 4-horned; placenta thready.

#### Phenology.

Old fruits in November.

#### Etymology.

The specific epithet is based on Ba Na hills, the type locality of this species.

#### Distribution.

*Perilimnastesbanaensis* is currently only known from Ba Na Hills, Hòa Ninh, Đà Nẵng, Vietnam (Fig. 1). It occurs on damp slopes in forests often near streams, at 1,360 m elevation.

## Supplementary Material

XML Treatment for Perilimnastes multisepala

XML Treatment for Perilimnastes setipetiola

XML Treatment for Perilimnastes uniflora

XML Treatment for Perilimnastes banaensis

## ﻿Additional information

### Author ORCIDs

Jin-Hong Dai https://orcid.org/0000-0001-5069-6016

Truong Van Do https://orcid.org/0000-0002-0585-5513

Ying Liu https://orcid.org/0000-0003-0613-837X

### Data availability

All of the data that support the findings of this study are available in the main text or Supplementary Information.

## Supplementary materials

Supplementary material 1 Supplementary data Data type docx Explanation note **table S1.** Comparison of *Perilimnastesmultisepala* and related species. The name of the new species is indicated in bold; **table S2.** Comparison of *Perilimnastesbanaensis*, *P.setipetiola*, *P.uniflora*, and related species. The names of the new species are indicated in bold. https://binary.pensoft.net/file/929976This dataset is made available under the Open Database License (http://opendatacommons.org/licenses/odbl/1.0/). The Open Database License (ODbL) is a license agreement intended to allow users to freely share, modify, and use this Dataset while maintaining this same freedom for others, provided that the original source and author(s) are credited.Jin-Hong Dai, Truong Van Do, Ying Liu
