# Supplementary material for: ﻿Four new species of Perilimnastes (Sonerileae, Melastomataceae) from Vietnam
Source: PhytoKeys. 2023 Nov 3;235:1–19. doi: 10.3897/phytokeys.235.112133 (PMC10638611; doi:10.3897/phytokeys.235.112133)
Supplement: Supplementary material 1 — Supplementary data [file phytokeys-235-001_article-112133__-s001.docx]

**Table S1.** Comparison of *Perilimnastes multisepala* and related species. The name of the new species is indicated in bold.

| Species | Habit | Crystal type | Indumentum of stems and petioles | Leaf blade | Inflorescence | Pedicels | Calyx lobes | Petals | Anthers | Habitat | Geographical distribution |
| --- | --- | --- | --- | --- | --- | --- | --- | --- | --- | --- | --- |
| ***P.* *multisepala*** | Shrubs to 80 cm tall | Druses | Glabrescent when mature | Obovate-lanceolate, oblong-lanceolate to oblanceolate, 2.4–8 × 0.7–2.4 cm | Terminal, 1- or 2-flowered, rarely 3- flowered | 1–2 mm long in flower and fruit | 4–8, linear | 28 × 9 mm, apex long acuminate | Yellow | On rocks along stream in forests, at 574 m | Đại Lộc, central Vietnam |
| *P. setotheca* | Shrubs to 100 cm tall | Druses | Glabrous when mature | Oblong-lanceolate,  elliptic, or obovate, 10–20 × 3–8 cm | Terminal, 3 to more than 20-flowered | 9 mm in flower, 25 mm in fruit | 4, long triangular | 14 × 8 cm, apex short acuminate | Purplish red | Damps places in forests, on slops or along stream, 300–550 m | Guangxi and Guangdong, China, and northern Vietnam |
| *P. fruticosa* | Shrubs to 100 cm tall | Druses | Glabrous or with minute brown glands | Elliptic-lanceolate, 2.8–7.5 (–14) × 0.6–2.8(–4.2) cm | Terminal, 1–4-flowered | 5 mm long in flower and 17 mm long in fruit | 4, linear triangular | 8.5–16 × 3.5–5 mm, apex acuminate | Yellow | Along stream in forests, at 700–2,000 m | Pahang, Malaysia |
| *P. stenophylla* | Shrubs to 80 cm tall | Druses | Glabrous | Oblong-lanceolate, 5–10 × 1–2.3 cm | Terminal, 2–3-flowered | 5–8 mm long in flower and 15 mm long in fruit | 4, narrowly triangular | 12 × 6 mm, apex short acuminate | Purple | Among rocks along stream in forests, at 500–920 m | Hainan Island, China |

**Table S2.** Comparison of *Perilimnastes banaensis*, *P. setipetiola*, *P. uniflora*, and related species. The name of the new species is indicated in bold.

| Species | Habit | Crystal type | Indumentum of stems and petioles | Leaf blade | Inflorescence | Pedicels | Calyx lobes | Petals | Anthers | Habitat | Geographical distribution | |
| --- | --- | --- | --- | --- | --- | --- | --- | --- | --- | --- | --- | --- |
| ***P.* *banaensis*** | Shrubs to 60 cm tall | Raphides | Villous with appressed, brown hyaline uniseriate hairs | Elliptic, 5.5–13 × 2.5–6.5 cm | Terminal, sessile, 2–7-flowered | 5–13 mm long in fruit | Unknown | Unknown | Unknown | On damp slopes near stream in forests, at 1,360 m | Đà Nẵng, Central Vietnam |  |
| ***P. setipetiola*** | Shrubs 40–120 cm tall | Raphides | Stems pubescent with stellate hairs when young, petioles hispid with long bristles | Broadly elliptic to elliptic, 5.6–15 × 1.9–6.4 cm | Terminal and axillary, peduncle 0–2 mm long, 2–11-flowered, | 8–13 mm long in flower and 16–25 mm in fruit | 4, triangular-ovate | 10 mm long, apex acute | Pink | Damp places in forests, at 1,500–1,700 m | Đà Lạt, southern Vietnam |  |
| ***P. uniflora*** | Shrublets or woody herbs to 30 cm tall, prostrate at middle and lower parts | Raphides | Glabrescent when mature | Obovate to obovate-lanceolate, 4.2–9.5 × 1.3–3.4 cm | Terminal, solitary flower | 2 mm long in flower, 4 mm in fruit | 4, linear | 11–13 × 4–6 mm, apex acute to short acuminate | Pink | On rocks along stream in forests, at 1,360 m | Đà Nẵng, Central Vietnam |  |
| *P. dispar* | Herbs to 40 cm tall, prostrate and rooting  basally | Raphides | With pale brown hyaline hairs | Unequal in a pair, elliptic,  larger ones 4–8 × 1.3–3 cm, smaller ones 3–11 × 1–6.5 mm | Terminal or axillary, solitary flower | Sessile or subsessile | 4, narrowly triangular | 7–10 × 6–6.5 mm, apex acute | Yellow | Damp places in forest, at 1,000–2,000 m | Borneo |  |
| *P. elliptica* | Herbs to 45 cm high | Raphides | With pale brown hyaline hairs | Elliptic or obovate, 4–  15.5 × 2.2–7.8 cm | Subterminal or axillary, peduncles 0–10 mm long, many- or few-flowered, rarely solitary | 16–31 mm long in flower, to 38 mm long in fruit | 4, broadly attenuate to subligulate | 11–16 × 6.5–10 mm, apex acute | Yellow | On slopes or along streams in forests, at 1,250–1,850 m | Borneo |  |
| *P. guillauminii* | Shrubs, height unknown | Raphides | With ascending to patent long bristles | Elliptic, 2.5–3.5(–6) × 0.7–l.4 cm | Terminal, solitary or in pairs | 3.5 mm in flower, 16 mm in fruit | 4, ligulate | 10 × 7 mm, apex acute | Pink | Unknown | Southern Vietnam |  |
| *P. setotheca* | Shrubs to 100 cm tall | Druses | Glabrous when mature | Oblong-lanceolate,  elliptic, or obovate, 10–20 × 3–8 cm | Terminal, subsessile or peduncle up to 3.5 cm long, 3 to more than 20-flowered | 9 mm in flower, 25 mm in fruit | 4, long triangular | 14 × 8 cm, apex short acuminate | Purplish red | Damps places in forests, on slops or along stream, 300–550 m | Guangxi and Guangdong, China, and northern Vietnam |  |
| *P. ovalifolia* | Shrubs 90-250 cm tall | Raphides | Stems densely retrorse hirsute, glabrescent, and petioles densely hirsute to setose, sometimes with brown hyaline hairs | Ovate to elliptic, 5.5–16 × 2.3–7.8 cm | Terminal,  peduncle 1–1.5(–3) cm long, 3–30-flowered | 8–13mm long in flower, to 20 mm in fruit | 4, triangular | 8.5–14 × 5–6.5 mm, apex acute | Purple | Damp places in forests, often on steep slopes, at 700–1,800 m | Guangxi and Guangdong, China, and northern Vietnam |  |
| *P. rupicola* | Woody herbs 8–60 cm tall | Raphides | With pale brown hyaline hairs | Narrowly elliptic, 3.5–7 × 0.4–1.8 cm | Terminal, sessile,  few-flowered | 9 mm long  in flower, to 22 mm long in fruit | 4, subligulate | 10–13 × 4–4.5 mm, apex short acuminate | Yellow | On rocks in river bed in forests, at 800 m | Borneo |  |
